# Use of Gold Nanoparticles to Detect Water Uptake in Vascular Plants

**DOI:** 10.1371/journal.pone.0114902

**Published:** 2014-12-11

**Authors:** Bae Geun Hwang, Sungsook Ahn, Sang Joon Lee

**Affiliations:** 1 Center for Biofluid and Biomimic Research, Department of Mechanical Engineering, Pohang University of Science and Technology (POSTECH), San31, HyojaDong, Nam-Gu, Pohang, Gyeongbuk, 790–784, South Korea; 2 Center for Biofluid and Biomimic Research, Pohang University of Science and Technology (POSTECH), San31, HyojaDong, Nam-Gu, Pohang, Gyeongbuk, 790–784, South Korea; University of Milano-Bicocca, Italy

## Abstract

Direct visualization of water-conducting pathways and sap flows in xylem vessels is important for understanding the physiology of vascular plants and their sap ascent. Gold nanoparticles (AuNPs) combined with synchrotron X-ray imaging technique is a new promising tool for investigating plant hydraulics in opaque xylem vessels of vascular plants. However, in practical applications of AuNPs for real-time quantitative visualization of sap flows, their interaction with a vascular network needs to be verified in advance. In this study, the effect of AuNPs on the water-refilling function of xylem vessels is experimentally investigated with three monocot species. Discrepancy in the water uptakes starts to appear at about 20 min to 40 min after the supply of AuNP solution to the test plant by the possible gradual accumulation of AuNPs on the internal structures of vasculature. However conclusively, it is observed that the water-refilling speeds in individual xylem vessels are virtually unaffected by hydrophilically surface-modified AuNPs (diameter ∼20 nm). Therefore, the AuNPs can be effectively used as flow tracers in the xylem vessels in the first 20∼30 min without any physiological barrier. As a result, AuNPs are found to be useful for visualizing various fluid dynamic phenomena occurring in vascular plants.

## Introduction

Quantitative visualization of water-conducting pathways and sap flows inside xylem vessels is indispensable for understanding the biological features of vascular plants and examining the disputable mechanism of sap ascent [1], [2]. Such observation of vascular plants under in vivo conditions has been nearly impossible because of technological limitations despite its significance. One of the conventional approaches is to directly supply dye solution into the vascular network. The flow passages of the stained sap can be optically observed after sectioning the plant sample. Sano et al. utilized liquid nitrogen to stabilize dye spread at a specific instant by freezing the sample [3]. As a noninvasive bio-imaging technique, magnetic resonance imaging (MRI) has been used to visualize three-dimensional (3D) spatial distribution of water in thick, opaque plant samples [4], [5], [6]. To measure the dynamic movement of sap flow, synchrotron X-ray micro-imaging technique has been recently introduced as a noninvasive modality for observing opaque plant samples. Combination with modern imaging devices such as a charge-coupled device (CCD) camera enables to capture X-ray images with a micro-scale spatial resolution at several tens or hundreds frames per second (fps). This X-ray imaging technique has been successfully used to investigate morphological structures and hydrodynamic phenomena in the xylem vessels of vascular plants [7], [8], [9]. However this experimental technique has been applied only to excised plant samples by creating an artificial air/water interface through dehydration and rehydration process. The upward movement of sap is recognized by tracing the meniscus between air and sap liquid. Therefore, this technique is hard to obtain hydrodynamic information on the sap flow in intact plants. To resolve this problem, we have to seed tracer particles into the sap flow in xylem vessels. Particle image velocimetry (PIV) or particle tracking velocimetry (PTV) has become a reliable velocity field measurement technique in fluid-mechanical field [10]. For this kind of velocity field measurement, suitable flow tracers with good contrast against surrounding substances are required to extract the velocity field information as a function of time. In earlier X-ray PIV measurements, alumina particles [11] and silver-coated hollow glass [12], [13] are seeded to track various opaque fluid flows. However, they are not ideal tracers for real biofluid flows because they are not biocompatible or their specific weight slightly differs from the biofluids to be measured.

One of the promising tracers for the X-ray imaging experiments of biofluid flows is gold nanoparticle (AuNP). The X-ray absorption efficiency of gold (Au) is about 2.7 times higher (5.16 cm/g at 100 KeV) than that of the commonly used X-ray contrast agent iodine (1.94 cm/g at 100 KeV). The X-ray attenuation coefficient of Au is even 150-fold higher than that of soft tissue (0.169 cm/g at 100 KeV) [14]. In addition, AuNPs can be easily fabricated in various sizes and shapes, and their surface is modified with high versatility. This kind of feature is one of the great advantages of AuNPs as a tracing sensor, because conventional liquid-type X-ray contrast agents (such as iodine) do not provide discrete positional information in the X-ray images of biofluid flows [15].

AuNPs have been known as a biocompatible material [16]. However, their interactions with vascular bundles of plants are not yet fully understood. Especially, the uptake and accumulation processes of AuNPs and their biological impact on the morphological structure and biochemical properties of plants remain as open questions [17]. Therefore, the obstruction of AuNPs in the vascular function of test plants by clogging the internal pathways and the reliable working period of using AuNPs as flow-tracing sensor in xylem vessels of vascular plants should be verified in advance for practical applications of AuNPs to the real-time visualization of in vivo sap flow phenomena.

To apply this experimental methodology to track water movement in intact plants, AuNPs should be directly injected into their xylem vessels. For this purpose, a precise injection system equipped with a pressure control device and a micro-manipulator is required to be developed. Such a pressure-controlled injection of AuNP solution will be discussed later. In this study, we mainly focus on the feasibility of the surface-treated AuNPs flow-tracing particles in xylem vessels of three excised monocot leaf samples. We also focused on the clogging effect of AuNPs and the hydraulic resistance of sap flow in pipe-like xylem vessels in a relatively short period of time up to 30 min, rather than detailed plant physiology of intact plants. The effects of repeatedly supplying AuNPs solution on the refilling function of xylem vessels of three different plant samples were investigated, and the functional deterioration of AuNP as a reliable flow tracer of sap flow inside xylem vessels was determined as a function of time.

## Materials and Methods

### Plant models

Three different monocot species were tested as plant samples in this study. Five leaves 36–87 cm long were collected from four maize (*Zea mays L.*) samples 41–103 cm in height grown for 4–8 weeks in a cultivation room with room temperature, relative humidity, and photosynthetically active radiation (PAR) maintained at 25°C, 70%, and approximately 300 µmol m^−2^ s^−1^ (10 h a day), respectively. PAR was monitored using a PAR sensor (E90/Jauntering International Corp., Taipei, Taiwan). Bamboo and rice leaves were collected from field-grown plants. Eight rice (*Japonica, Oryza sativa L.*) leaves 31–43 cm long were collected from a seed bed grown for 10 weeks from germination. Six bamboo leaves 10–13 cm long were collected from a 1.5 m-high branch excised from a wild-type bamboo (*Bambusoideae*) about 5 m high. The cut end of the excised branch was immediately immersed in water after the excision. Specific permissions were not required for collecting the samples and the field studies did not involve endangered or protected species.

### Preparation of the surface-modified AuNPs

The AuNP of 20 nm in diameter was modified to have hydrophilic surface for enhancing biocompatibility, which was reported to show the optimum function in biological systems [18]. Detailed fabrication procedures are available in our previous study [15]. In brief, for the preparation of gold nanoparticles (AuNPs), gold chloride (III) trihydrate (HAuCl_4_⋅3H_2_O) was dissolved in DI Milli-Q water (1.0×10^−3^ mol/L) under refluxing. Sodium citrate tribasic dihydrate solution in DI water (4×10^−2^ mol/L) was added to the above solution. Reaction completion was detected by color changes from pale yellow to wine red. Boiling condition was further maintained for 15 min after the color change was completed and then cooled to room temperature. The AuNPs solution was dialyzed overnight against DI Milli-Q water to remove excess sodium citrate tribasic dihydrate. Thiol ligand was added at the second step. After the size of the AuNP was determined, 40 µL of 0.1 M ligand aqueous solution was added to the above aqueous solution to modify the surface properties of the citrate-covered AuNP. 2-mercaptoethanol (SH-CH_2_CH_2_OH) was added to the solution for hydrophilic surface-modification and stirred at room temperature or higher (between 50°C and 60°C) for 6 h to 12 h until no further color change was observed. The un-reacted residue ligands were minimized to less than 1 ppm by testing aliquot of the samples. The AuNPs solutions were dialyzed overnight against Milli-Q water for purification. The AuNPs solutions were placed on a typical copper grid and then dried under air at room temperature for transmission electron microscopy (JEOL Cs-corrected HR-TEM, JEM-2200FS). Gold cores with high electron density in each AuNPs were captured distinctively in TEM images. The average diameter of the AuNPs was controlled to be approximately 20 nm. The aqueous surface-modified AuNP stock solutions were adjusted to have a concentration of around 2.4×10^18^ AuNPs/m^3^ and the HAuCl_4_ concentration of 1.0×10^−3^ mol/L. The AuNPs solution was treated with a sonicator (JAC-2010 ULTRASONIC/KODO Technical Research, Hwaseong, South Korea) for 20 min in a glass vial not more than 5 h before each X-ray experiment.

### Synchrotron X-ray imaging

Synchrotron X-ray imaging technique was used to obtain phase-contrast images of water transport in the xylem vessels of living organisms. The X-ray imaging technique used for monitoring the sap flow is described in detail in our previous studies [7], [8], [9]. The present experiments were conducted at the 6D X-ray micro-imaging beamline (PLS-II) of Pohang Accelerator Laboratory (PAL). The X-ray image of the test sample was converted into visible light by passing through a scintillator crystal. A CCD camera (Vieworks VH-11MC) attached with a 10× objective lens was used to capture X-ray images in a field of view (FOV) of 3600×2400 µm^2^ with a spatial resolution of 0.9 µm/pixel at a frame rate of 5 fps. When a 5× objective lens was attached, FOV was broadened to 7200×4800 µm^2^ with a spatial resolution of 1.8 µm/pixels. The exposure time was regulated from 60 ms to 100 ms depending on the sample thickness and beam flux. A mechanical shutter and attenuating plates were utilized to minimize damage on the test sample by X-ray beam exposure.

**Figure 1:**
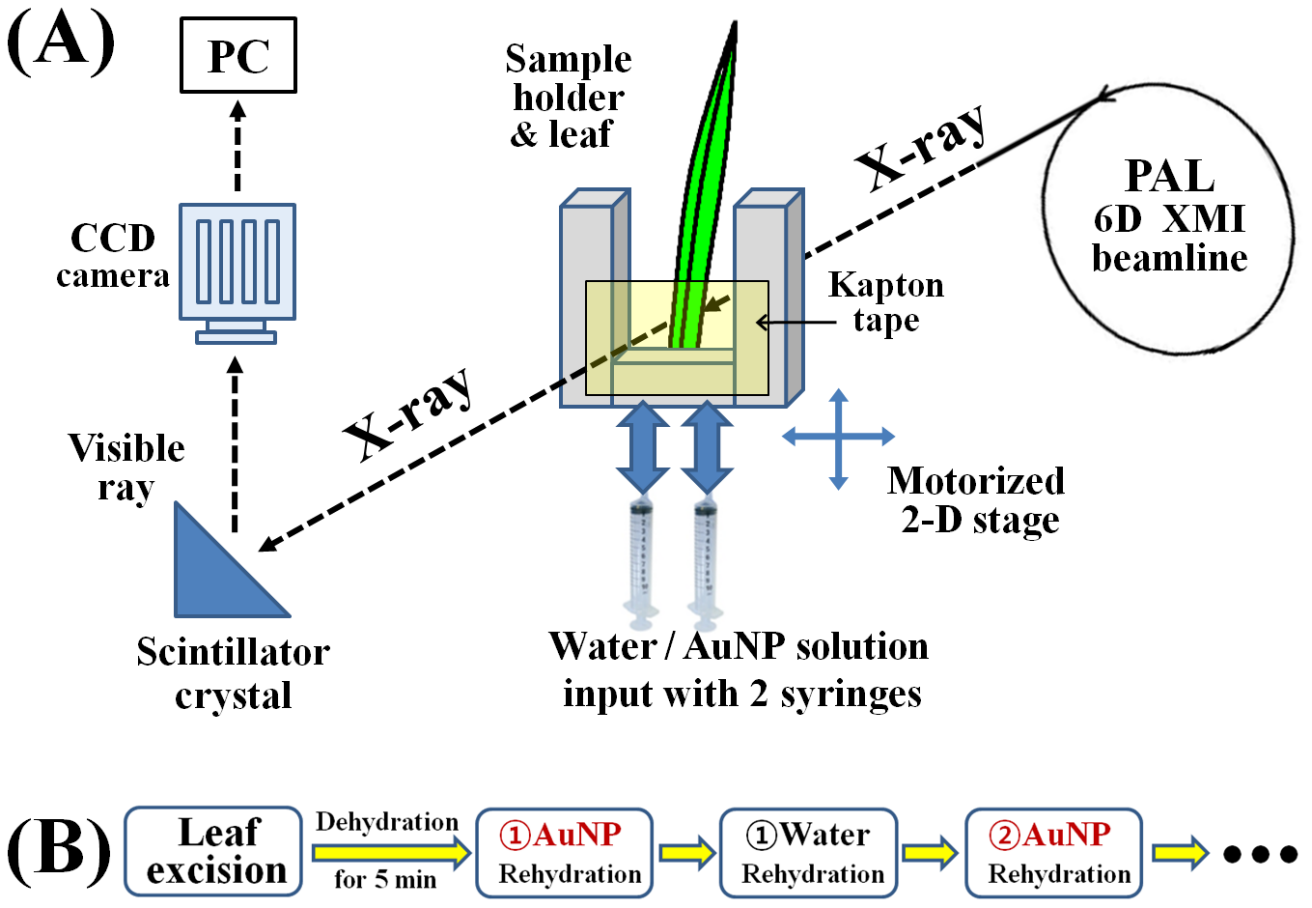
Experimental setup for synchrotron X-ray imaging and sample treatment. (A) Setup for visualizing the water-refilling process in 6D X-ray micro-imaging beamline of PAL. (B) Schematic description of alternative supplies of AuNPs solution and distilled water to the test sample. Each arrow between adjacent rehydration processes indicates 5 min dehydration in air.

A stable environmental condition is maintained in the experimental hutch of PAL during X-ray imaging. The temperature was controlled from 24°C to 26°C under a relative humidity of 31% to 60% according to the experimental condition. The PAR intensity was fixed at 35 µmol m^−2^ s^−1^. The plant leaves tested in this study were mostly excised 10 cm from the extremities, excluding three <12cm-long bamboo leaves excised at 8–9 cm. Then, the sample was vertically placed on a U-shaped sample holder affixed to a motorized 2D stage. The front and backside of the sample holder were sealed with Kapton tape to contain water and transmit an X-ray beam. The 2D stage was utilized to accurately position the test sample at the FOV or to trace the temporal displacement of the water meniscus (Fig. 1A). After dehydrating the excised sample for 5 min in air, the end cut of the sample was immersed in water for rehydration. Real-time phase-contrast X-ray images of the refilling process were consecutively recorded. After 5 min of continuous recording of the rehydration process, the leaf sample was then dehydrated for 5 min again by draining water from xylem vessels. The rehydration and recording procedure was repeated again after 5 min. To compare the refilling process of AuNP solution and pure water in the identical xylem vessels, the leaf samples were alternately rehydrated with AuNP solution and distilled water (Fig. 1B). The rehydration–dehydration cycle was continuously repeated until the water-refilling performance of the sample significantly decreased, usually up to four times.

### Evaluation of xylem-vessel functionality

We quantitatively analyzed two metaxylem vessels in a xylem bundle of monocot leaves that exhibited relatively uniform and active refilling during the repeated cycles. The functionality of each xylem vessel was evaluated from the variation in water-refilling speed, which was determined by the temporal displacement of a water column in the xylem conduit. The kinetic movements of the water columns were analyzed using the digital image processing software ImageJ.

**Figure 2:**
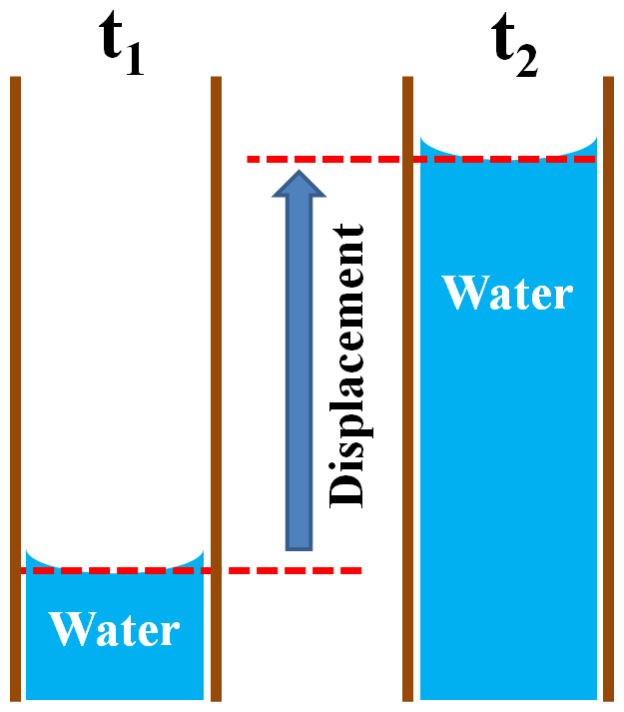
Schematic description of the method to measure the refilling speed of water uptake. Water-refilling speed is calculated by dividing the longitudinal displacement of air-water meniscus in an embolized xylem vessel at specific time t_1_ and t_2_, which indicates the height variation of the refilling water column, by the time interval (t_2_–t_1_).

A 10× objective lens was used for the detailed observation of maize leaves. Temporal movement of the water meniscus was continuously tracked by traversing the motorized 2D stage. The pixel coordinates of the center of each water–air meniscus in the phase-contrast X-ray images were successively obtained from the consecutively recorded X-ray images. The uprising speed of each water column was obtained based on the displacement of the corresponding pixel coordinates for a given time interval (Fig. 2). If the X-ray images were vibrating or multiple menisci were simultaneously moving, several distinctive structures in the xylem vessels were designated as fixed reference points to evaluate meniscus displacements using relative coordinates. The total sum of elapsed times of water stoppage at perforation plates were obtained from the temporal variations of water-refilling speed.

For rice and bamboo samples, a 5× objective lens was used, and FOV was fixed at the lowest part of the sample. The excised point was positioned at the bottom of the FOV, whose observable elevation was restricted almost to the vertical range of FOV (4.8 mm). When the vertical position of the refilling water column exceeded the observable range during image recording, the average water-refilling speed was calculated with the time elapsed to reach the top of FOV and the observable maximum displacement in height. Otherwise, it was evaluated from the longitudinal variation in the refilling water column during 5 min period of image recording.

### Water uptake volume measurement

In addition to the synchrotron X-ray imaging experiment, the uptake rates of AuNP solution and distilled water were quantitatively compared. Rice and bamboo leaves were collected from the same plants used for X-ray imaging experiments. They were excised 1 cm from the bottom. The top part of the vial used as a test chamber containing the tested AuNP solution or distilled water was sealed with parafilm in which a narrow slit was made. The test sample was inserted through the slit and immersed into the AuNP solution or distilled water immediately after excision. The total weight of the vial was measured at intervals of 10 min with an electronic scale (CAUW-220D/CAS, Seoul, South Korea). The portion of rice leaf exposed to air for transpiration was 15 cm long and 1.1 cm wide. In the case of bamboo, since multiple samples with various lengths ranging within 10–17 cm were used, the experimental data for each sample were normalized with the length exposed to air above the vial. The widths of the bamboo leaves tested in this study were relatively uniform (2–2.5 cm). During the experiment, the environmental condition was kept stable in a constant temperature and constant humidity facility whose room temperature, relative humidity, and PAR were fixed at 23°C, 48.5%, and approximately 30 µmol m^−2^ s^−1^, respectively.

## Results

### Effect of AuNPs on water-refilling in xylem vessels

**Figure 3:**
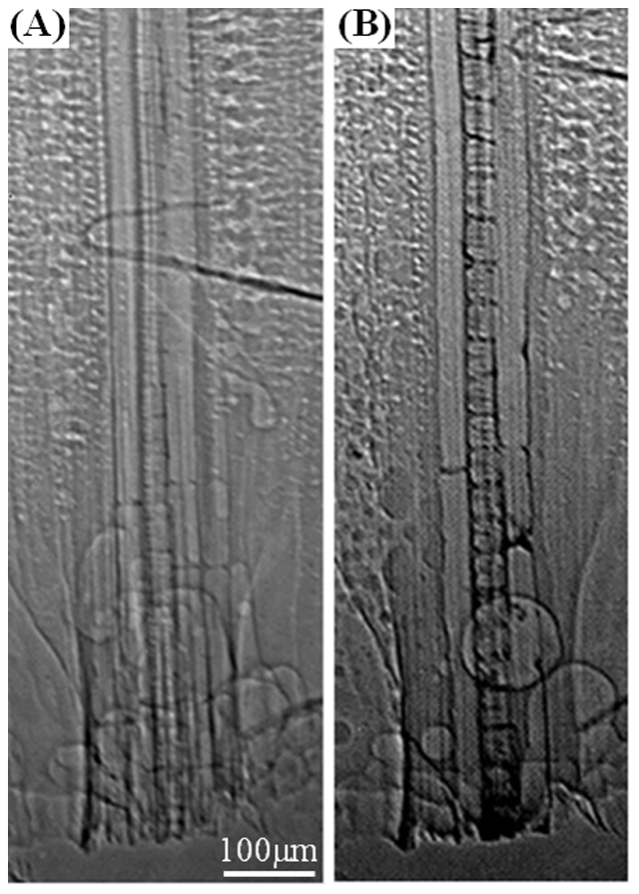
Typical X-ray images of xylem vessels in a maize leaf. Identical xylem bundle (A) before the first rehydration with AuNP solution and (B) after the first rehydration and dehydration cycle. Vessel wall is darkened by attached AuNPs.

The anatomical features of xylem vessels and their internal morphological structures are clearly distinguishable in previously captured X-ray images (Fig. 3A) and after the supply of AuNPs (Fig. 3B). Given that AuNPs are gradually stained on the surfaces of cell walls of xylem networks, the morphological structures of xylem vessels can be easily distinguished. This implies that AuNPs are actually entered into the xylem vessels during the rehydration of AuNPs solution, and some of them are remained inside the xylem vessels after dehydration. In most cases, the AuNP staining induces xylem vessels to exhibit distinguishable changes after the 1^st^ supply of AuNPs solution. However, afterward continued supplies of AuNPs solution do not bring about drastic intensification on the portions once stained in X-ray images. Meanwhile, the supply of distilled water also do not dilute the stained portions noticeably. These results imply that the interaction between AuNPs and the stained xylem vessel structures is strong enough to keep the stained AuNPs from washing out by water stream.

**Figure 4:**
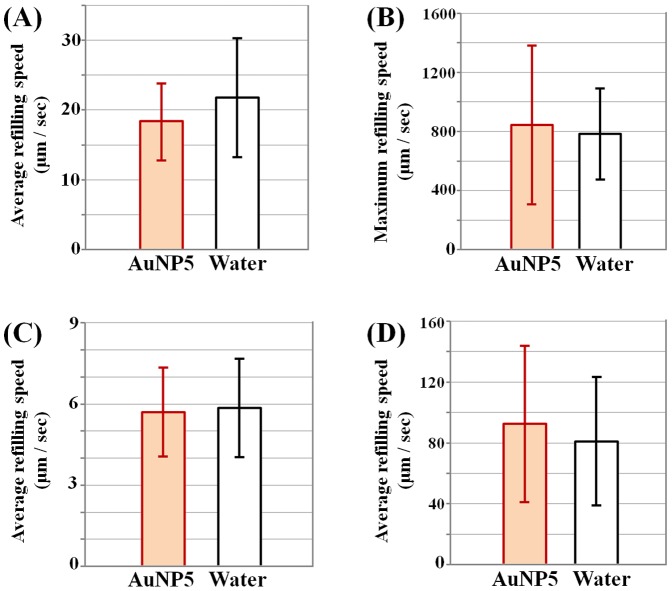
Comparison of average water-refilling speeds of AuNP solution and distilled water. (A) Average and (B) maximum water-refilling speed in maize leaves (*n* = 47). (C) Average water-refilling speed in rice leaves (*n* = 78) and (D) in bamboo leaves (*n* = 42).

The water-refilling processes in the dehydrated xylem vessels are similar to the behaviors observed in our previous studies by using the same technique [7], [8], [9], whether AuNP solution or distilled water is used in the rehydration process. Fig. 4 compares the water-refilling speeds measured in xylem vessels of maize (Figs. 4A, B), rice (Fig. 4C), and bamboo (Fig. 4D) leaves. Despite the relatively large variation in the values, the average water-refilling speeds for AuNPs solution and distilled water are nearly identical. This result indicates that the effect of AuNP supply on the water-refilling process in xylem vessels is insignificant from the viewpoints of complexity and diversity of biological systems in nature.

**Figure 5:**
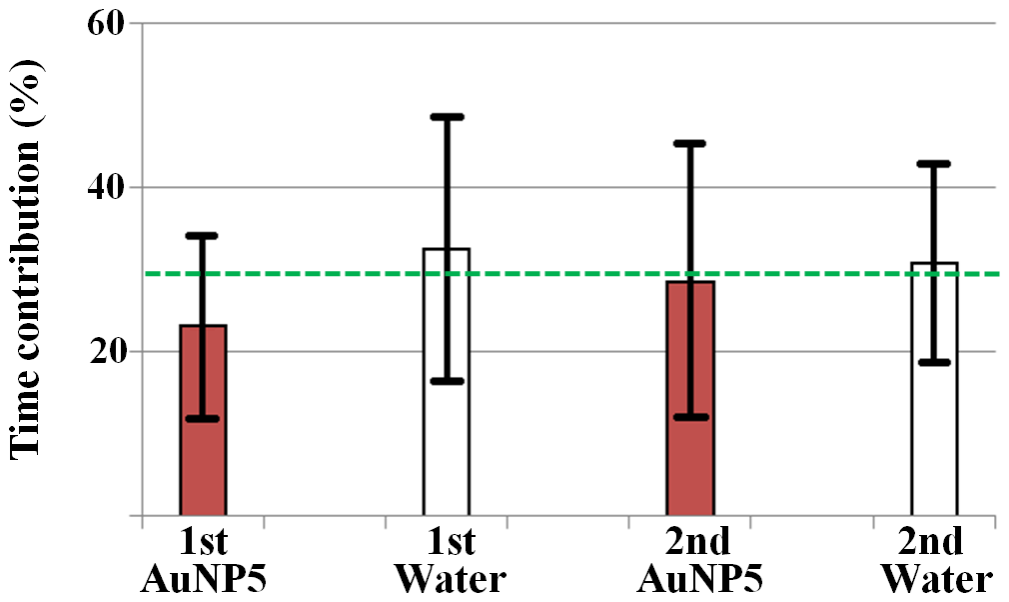
Contribution of water stoppage time at perforation plates. The ratio of water stoppage time at perforation plates to the total time elapsed for water-refilling in the xylem vessels of maize leaves. The dotted line represents the average time contribution of 28.9%. Vertical bars represent the minimum and maximum values.

Perforation plates are the perforated end wall of vessel elements, which often work as a hydraulic valve in water-refilling process. Fig. 5 compares the contribution of the time spent for water stoppage at perforation plates to the total time elapsed in the water-refilling process of xylem vessels of maize leaves. The time contributions of perforation plates at four different stages throughout the experiment have the mean value of 28.9%.

### Validation of water uptake volume

**Figure 6:**
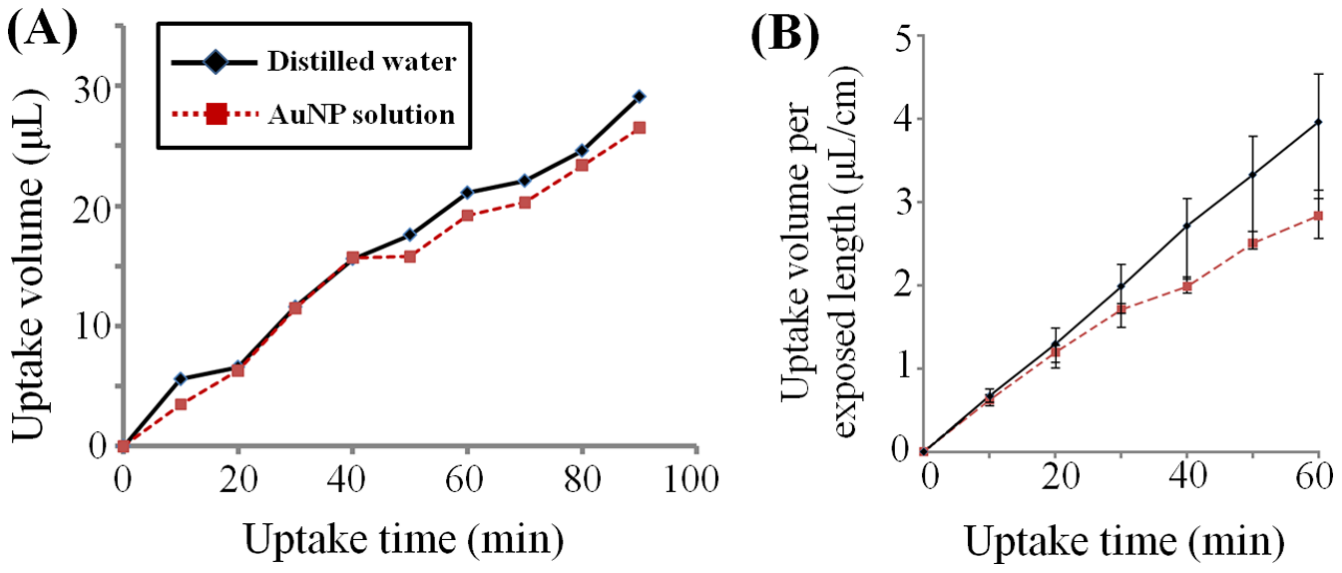
Comparison of water uptake volume for AuNP solution and distilled water. (A) Comparison of water uptake volume for a rice leaf. (B) Water uptake volume normalized by the exposed length of bamboo leaves (*n* = 3 for each). Variation in average water volume with the maximum and minimum value presented.

In this study, direct evaporation from the vial was blocked. Therefore, water transport through xylem vessels and the evaporation at stomata may exclusively contribute to the loss of water inside the vial used as a test chamber. Based on this aspect, the quantity of water uptake was estimated from the weight loss of the vial. Given that the AuNPs entered into the vascular network cannot evaporate through stomata, the weight loss in the vial containing AuNPs solution can be regarded as the escape of pure water. Fig. 6 compares the variations in uptake volumes of AuNPs solution and distilled water. Their variation trends are similar in the early stage. However, deviation started to appear at about 20∼40 min later. The uptake rate of AuNPs solution is decreased to about 31% in the bamboo leaves at the elapsed time of 60 min.

## Discussion

The surface-modified AuNPs used in this study are demonstrated to be suitable flow-tracing particles for measuring sap flows in vascular plants without noticeable decrement in xylem-vessel functionality within the initial half hour under the similar experimental condition used in this study. If this experimental methodology was applied to intact plants, the difference in environmental conditions such as uptake water flow rate may somewhat affect the reliable working period of AuNPs as flow tracers.

Perforation plate is the perforated end wall between adjacent vessel elements. In the pathways of xylem vessels, perforation plates are a kind of physical barriers to sap flow. They are constituted of hydrolyzed cell walls composed of cellulose microfibril networks. The shape of perforated opening includes single rim, scalariform, reticulate and other variations of small pores, some of which are small enough to regulate the transport of sap flow which accompanies embolized gas bubbles, especially in the water-refilling process. The physicochemical conditions of the perforation plates and pit membranes, such as porosity and pore size, are mediated by the ionic compositions of the xylem sap. Their variations are one of the essential clues to understanding the entire metabolism of plants [19], [20]. In general, contrast agents need to be large in size to get sufficient contrast from the transmitted X-ray. Meanwhile, their size has to be sufficiently small to pass through the pores of membranes [17]. If a cluster of AuNPs clogs the sieve-like pores of perforation plates, the clogging effect becomes more devastating, compared to the accumulation of AuNPs on the walls of xylem vessel conduits, whose inner diameters are mostly larger than 20 µm. Therefore, in the beginning stage of this study, we suspected the clogged pores of perforation plates are the major bottleneck of tracer particles in the water-refilling process of vascular plants. However, experimental results show that the average water-refilling capability is not so noticeably obstructed during the time period tested in this study (Fig. 4). Moreover, the ratio of the time of water stoppage at perforation plates to the total time elapsed in the water-refilling process appears to be kept around the average value of 28.9% at four different stages throughout the experiment (Fig. 5). This finding indicates that the supply of AuNPs does not induce any drastic change in the function of perforation plates to regulate sap flow in xylem vessels at least for the tested time period. This finding also demonstrates that the clogging of clustered AuNPs at perforation plates does not have serious influence on the water-refilling process at least during the tested time period. However, very small pores of pit membranes could be easily and quickly occluded by AuNPs. This clogging of pit membrane may affect the radial transport of water between adjacent xylem conduits. The effect of AuNPs exposure on pit membranes and radial water transport should be considered in future study.

The water uptake volume is compared to estimate the working period of AuNP as suitable tracer particles of sap flow inside the xylem vessels of vascular plants. The amounts of water uptake and transpired water can be used to evaluate xylem vessel functionality. In this study, the water uptake volumes of AuNP solution and distilled water starts to show deviation after roughly 20 min to 40 min later for the monocot species tested in this study (Fig. 6). This finding implies that the xylem vessel functionality is nearly maintained without noticeable variation in the initial half an hour after AuNP supply. During synchrotron X-ray imaging experiments, the xylem vessels of test samples are directly in contact with AuNPs solution for about 10 min to 15 min in total when the dehydration and rehydration cycle is repeated two or three times. Therefore, the conservation of xylem-vessel functionality well coincides with the results of water uptake volume variation.

Weitz et al. [21] proposed a clogging model based on successive buildup of particles through irreversible sticking events on the pore. In their model, the number of particles required to clog a pore N* is given as: 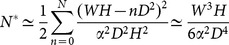
where *W* and *H* are the width and height of the pore, *D* is the particle size, and *α* is defined as *ε*/*D*. The sticking area *ε* is the characteristic distance at which particles are sticked to the pore irreversibly by attraction force. The minimum time required to clog a pore of 3 µm in diameter in 25 µm xylem vessel is estimated to exceed 4 h for *ε* = 10 nm, based on the assumption of a hypothetical sieve-like perforation plate. As the sticking area *ε* increases to 30 nm, the expected clogging time is decreased to 28 min, which is similar scale as the present experimental result (20∼40 min). If AuNPs of 20 nm are aggregated into cluster, the clogging time would be decreased. For example, AuNP cluster of *D* = 2 µm will take about 1 h to clog the pore for *ε* = 20 nm. Therefore, the aggregation and attraction characteristics of AuNPs to xylem vessel structures are critical to control the time period during which AuNPs can be used effectively to detect xylem sap flows.

In conclusion, at least in the initial 20∼30 min after the supply, AuNPs can be effectively used as tracer particles suitable for the quantitative visualization of sap flows in xylem vessels of the vascular plants by using synchrotron X-ray imaging technique.
